# Src Kinase and Syk Activation Initiate PI3K Signaling by a Chimeric Latent Membrane Protein 1 in Epstein-Barr Virus (EBV)+ B Cell Lymphomas

**DOI:** 10.1371/journal.pone.0042610

**Published:** 2012-08-03

**Authors:** Olivia Hatton, Stacie L. Lambert, Sheri M. Krams, Olivia M. Martinez

**Affiliations:** 1 Program in Immunology, Stanford University School of Medicine, Stanford, California, United States of America; 2 Department of Surgery/Division of Abdominal Transplantation, Stanford University School of Medicine, Stanford, California, United States of America; Karolinska Institutet, Sweden

## Abstract

The B lymphotrophic γ-herpesvirus EBV is associated with a variety of lymphoid- and epithelial-derived malignancies, including B cell lymphomas in immunocompromised and immunosuppressed individuals. The primary oncogene of EBV, latent membrane protein 1 (LMP1), activates the PI3K/Akt pathway to induce the autocrine growth factor, IL-10, in EBV-infected B cells, but the mechanisms underlying PI3K activation remain incompletely understood. Using small molecule inhibition and siRNA strategies in human B cell lines expressing a chimeric, signaling-inducible LMP1 protein, nerve growth factor receptor (NGFR)-LMP1, we show that NGFR-LMP1 utilizes Syk to activate PI3K/Akt signaling and induce IL-10 production. NGFR-LMP1 signaling induces phosphorylation of BLNK, a marker of Syk activation. Whereas Src kinases are often required for Syk activation, we show here that PI3K/Akt activation and autocrine IL-10 production by NGFR-LMP1 involves the Src family kinase Fyn. Finally, we demonstrate that NGFR-LMP1 induces phosphorylation of c-Cbl in a Syk- and Fyn-dependent fashion. Our results indicate that the EBV protein LMP1, which lacks the canonical ITAM required for Syk activation, can nevertheless activate Syk, and the Src kinase Fyn, resulting in downstream c-Cbl and PI3K/Akt activation. Fyn, Syk, and PI3K/Akt antagonists thus may present potential new therapeutic strategies that target the oncogene LMP1 for treatment of EBV+ B cell lymphomas.

## Introduction

EBV is a γ-herpesvirus that has infected >90% of the world's population. In immunocompetent hosts EBV infection is generally asymptomatic, although some adolescents develop self-limiting infectious mononucleosis [1]. Nevertheless, EBV is also linked to a variety of malignancies including Hodgkin's disease, Burkitt's lymphoma, B cell and NK cell lymphomas, and nasopharyngeal carcinoma. EBV has potent transforming ability, as infection of B lymphocytes results in the generation of immortalized lymphoblastoid cell lines (LCL) *in vitro* and autonomously proliferating lymphoblasts *in vivo*. Immunocompetent hosts readily control the expansion of EBV+ lymphoblasts through vigorous anti-viral T cell immunity. However, in immunocompromised individuals, like those infected with HIV or immunosuppressed transplant patients, EBV-infected lymphoblasts can give rise to AIDS-related lymphomas or post-transplant lymphoproliferative disorder (PTLD), respectively [2].

Latent Membrane Proteins 1 and 2a (LMP1 and LMP2a) are the two major signaling proteins of EBV, as they act as constitutively active mimics of B cell signaling molecules. LMP2a is a functional B cell receptor (BCR) mimic that propagates signals through spleen tyrosine kinase (Syk). LMP2a-derived signals function to sustain survival [3], [4] and maintain viral latency, in part by blocking BCR triggering [5], [6]. The major oncogene of EBV, LMP1, is a functional CD40 mimic. Interactions of cellular tumor necrosis factor (TNF) receptor-associated factor (TRAF) adaptor proteins, including TRAF1 [7], [8] and TRAF3 [7], [9], with the LMP1 C-terminal tail signaling domains, carboxy-terminal activating region 1 and 2 (CTAR1 or CTAR2), initiate signal transduction through a variety of pathways including the p38 [10], [11], Erk [12], [13], and JNK [14], [15] MAPK and NF-κB [16], [17] pathways.

Recent studies have also implicated LMP1 in the activation of the PI3K/Akt pathway. Given its ability to regulate cell survival and growth [18], it is unsurprising that deregulated PI3K activation is associated with a number of malignancies [19]. Activation of PI3K/Akt signaling by LMP1 results in secretion of the growth factor IL-10 in B cells [20] and is essential for the survival of LMP1-transformed Rat-1 cells [21]. Finally, inhibition of PI3K/Akt with the small molecule inhibitor LY294002 reduces proliferation and induces apoptosis of EBV+ PTLD derived B cell lines [22]. PI3K/Akt activation by LMP1 requires the CTAR1 domain [23], but the specific mechanisms by which LMP1 activates PI3K/Akt signaling remain to be determined. In the case of membrane-based receptors, Src family kinases and Syk are involved in the initial recruitment of the p85α subunit of PI3K [24]. Whether LMP1 signaling through PI3K/Akt involves the recruitment of similar helper proteins is unknown.

Syk signaling was initially believed to be restricted to classical immunoreceptors of the adaptive immune response, such as the BCR and Fc receptors (FcR) [25]. These receptors associate with signaling adaptors containing a short, tyrosine-containing peptide sequence known as the immunoreceptor tyrosine-based activation motif (ITAM) [26]. Receptor ligation results in phosphorylation of the two ITAM tyrosines by the Src tyrosine kinases. Syk is recruited to, and activated by, phosphorylated ITAMs. Recent studies revealed a requirement for Syk in several innate immune functions, such as detection of fungi and tissue damage [27], and certain non-immune functions, including vascular development [28] and bone metabolism [29]. In many of these cases, classes of receptors that do not contain conventional ITAMs, including C-type lectins [30] and integrins [31], activate Syk. Some C-type lectins, like the fungal recognition receptor Dectin-1/CLEC7A [32], contain a singular Y*XX*L motif (a ‘hemITAM’). Integrins, in contrast, do not contain either an ITAM or hemITAM, yet Syk-deficient monocytes [33], neutrophils, macrophages [34], osteoclasts [35], and platelets [36] display defective integrin signaling. These observed non-canonical Syk activation systems raise the possibility that other non-ITAM containing receptors may utilize Syk to coordinate downstream signaling pathway activation.

In the present study, we aimed to investigate how LMP1 activates PI3K. Given the recent finding that Syk can be activated in non-canonical fashion and evidence of Syk participation in PI3K activation, we asked if Syk and Src family kinases are activated in EBV+ B cell lymphomas and are required for activation of PI3K by LMP1. Using small molecule inhibition and siRNA strategies in human B cell lines expressing a signaling-inducible LMP1 protein (NGFR-LMP1), our results support the requirement for Syk and the Src family kinase Fyn in PI3K activation by LMP1.

## Methods

### Reagents

Streptavidin and antibodies to β-actin and NGFR (clone 20.4) were from Sigma (St. Louis, MO, USA). Biotinylated anti-NGFR was from Chromaprobe (Maryland Heights, MO, USA). Secondary antibodies, including PE-conjugated goat anti-mouse IgG, unconjugated goat-anti-mouse IgG, HRP-conjugated polyclonal goat anti-rabbit, HRP-conjugated polyclonal donkey anti-mouse, and Cy3-conjugated donkey anti-mouse antibodies were from Jackson Immunoresearch Labs, Inc (West Grove, PA, USA). Anti-LMP1 antibodies (clone CS.1-4) and isotype control antibodies were obtained from Dako (Carpinteria, CA, USA). Anti-LMP2a (clone 14B7) and rabbit anti-rat secondary antibodies were from Abcam (Cambridge, MA, USA). Anti-TRAF3 (H-20), anti-phospho-Erk (Tyr^204^), anti-Erk, and anti-P38 were from Santa Cruz Biotechnology (Santa Cruz, CA, USA). Antibodies to Akt, phospho-Akt (Ser^473^), c-Cbl, phospho-c-Cbl (Tyr^700^), JNK, phospho-JNK (Thr^183^/Tyr^185^), BLNK, phospho-BLNK (Tyr^96^), Syk, and phospho-Syk (Tyr^525/526^) were from Cell Signaling Technology (Danvers, MA, USA), as was anti-phospho-Src family (Tyr^416^), a rabbit polyclonal antibody that recognizes phosphorylated Src, Lyn, Fyn, Lck, Yes, and Hck. Anti-phospho-P38 (Thr^180^/Tyr^182^), anti-Fyn (mouse mAb 25/Fyn) and anti-Lyn (mouse mAb 42/Lyn), PE-conjugated ICAM-1 (CD54), and streptavidin-PE were from BD Pharmingen (San Diego, CA, USA). F(ab)_2_ fragments of anti-human IgM were from Invitrogen (Carlsbad, CA, USA).

### Cell Lines

The Burkitt's lymphoma line BL41 and its EBV-infected counterpart BL41.B95 were kindly provided by Dr. Elliot Kieff (Harvard Medical School) [37], [38]. BL41.NGFR-LMP1 (clone 1) expresses the chimeric NGFR-LMP1 molecule in the parental BL41 line; the generation of this line by our lab was described previously [20]. Cell lines AB5, JB7, and JC62 were generated in our lab as previously described and are spontaneously derived LCL grown from peripheral blood or lymph nodes of patients diagnosed with EBV+ PTLD [39]. All cell lines were maintained in a 5% CO_2_ humidified 37°C incubator in RMP1 1640 supplemented with 10% heat-inactivated FCS (Serum Source International, Charlotte, NC, USA), and 50 units/mL penicillin-streptomycin (Invitrogen). Growth media for BL41.NGFR-LMP1 was additionally supplemented with geneticin (Sigma). Prior to signaling studies, cells were grown overnight with reduced (4%) FCS.

### Flow Cytometry

For cell surface staining, 1×10^6^ cells were washed with cold FACS buffer (PBS, 1% BSA, 0.02% sodium azide) and incubated on ice for 30 minutes with 1 µg of primary antibody, washed, then incubated 30 minutes on ice with 0.4 µg of the fluorophore-conjugated secondary antibody. Stained cells were then washed and analyzed on a Becton Dickinson FACScan using Cellquest software.

### NGFR-LMP1 and BCR Crosslinking

NGFR-LMP1 expressing cells were resuspended at 5–10×10^6^ cells/mL in RPMI with 4% FCS. Cells were then stimulated as previously described [20] through the addition of unconjugated mouse anti-NGFR followed goat anti-mouse F(ab)_2_ for the indicated amount of time at 37°C. To initiate BCR signaling, cells were resuspended at 1×10^6^ cells/mL in RPMI with 4% FCS, and triggered to signal with 12 µg F(ab)_2_ fragments of anti-IgM per 10^6^ cells for the indicated amounts of time.

### Immunofluorescent Staining

Cells of interest were plated on 8-well LabTek II chamber slides (Nalge Nunc, Rochester, NY, USA) coated with poly-d-lysine (Sigma) at a concentration of 100,000–150,000 cells per well in growth medium for a minimum of 30 minutes in a 5% CO_2_ humidified 37°C incubator. Immunofluorescent staining was performed as previously described [40], [41] with the following antibody conditions: anti-LMP1 (Dako, 1∶250), donkey anti-mouse Cy3 (Jackson Immunoresearch, 1∶500). Slides were examined using a Zeiss 510 Meta microscope.

### Inhibition of Signal Transduction Pathways

The following inhibitors were used to analyze the effect of cellular signaling pathways on phosphorylation events and IL-10 production: PP2 (Src family kinase inhibitor, 5 µM) or R406 (Syk inhibitor, 0.5 µM). Inhibition of these pathways and storage of inhibitors was done as previously described [20]. All inhibitors were obtained from Calbiochem (Gibbstown, NJ USA), except R406, the active component of fostamatinib (Rigel, South San Francisco, CA, USA and AstraZeneca, London, UK). The appropriate concentration of inhibitor for each cell line was determined in preliminary experiments based on the ability to specifically block phosphorylation of the relevant signaling molecule without inducing cell death or toxicity.

### IL-10 ELISA

IL-10 levels were determined as previously described [20]. IL-10 produced per million cells was calculated based on live cell counts, as determined by trypan blue exclusion, at the time that cytokine-containing supernatants were harvested.

### Immunoprecipitation and Western Blot Analysis

Western blot analysis was performed as previously described [20]. For immunoprecipitation, lysates were pre-cleared with GammaBind G sepharose (Amersham, Piscataway, NJ, USA) for 1 h at 4°C and immunoprecipitated with 1 µg/10^7^ cells of the indicated antibody or an isotype control overnight at 4°C. Immune complexes were pulled down with GammaBind G sepharose, dissociated with 2× reducing sample buffer, and separated by SDS-PAGE prior to transfer to nitrocellulose membranes.

### RNA Interference (RNAi)

Fyn validated Stealth RNAi, Stealth Select Syk RNAi, and Stealth GC-matched negative control RNAi duplexed oligoribonucleotides were obtained from Invitrogen. BL41.NGFR-LMP1 cells (2–4×10^6^) were transfected with 100–200 nmol RNAi using the Amaxa Nucleofector 2 in 100 µl of cell line nucleofector kit V solution according to the manufacturer's instructions. A FITC-conjugated control RNA oligonucleotide confirmed transfection efficiency of at least 90% at 24 hours. Assays were initiated at 96 hours, with knockdown confirmed at this time point in cellular lysates. Western blotting of cellular lysates consistently demonstrated >80% specific knockdown of expression of the targeted gene compared to β-actin control.

### Quantitative real time PCR (qRT-PCR)

Total mRNA was isolated using TRIZOL reagent (Invitrogen) and cDNA was synthesized with an oligo(dT) primer (Invitrogen), followed by quantitative real time RT-PCR on a Stratagene Mx3000P using SYBR Green PCR master mix and primers for human IL-10 and GAPDH as previously described [20]. IL-10 was normalized to GAPDH for each sample.

## Results

### Membrane Expression of Native LMP1 and Chimeric NGFR-LMP1 in B Cell Lymphomas

**Figure 1:**
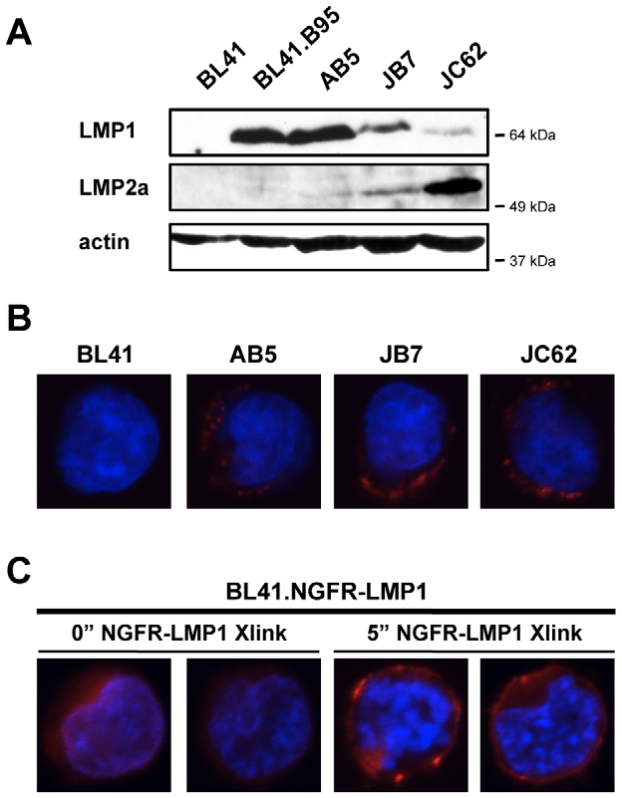
Membrane Expression of Native LMP1 and Chimeric NGFR-LMP1 in B Cell Lymphomas. *(A)* Cell lysates were separated by SDS-PAGE, transferred to nitrocellulose and probed with antibodies against LMP1, LMP2a, and actin as a loading control. For all western blots, the closest molecular weight marker (e.g. 64 kDa) to the protein of interest is indicated to the right of each blot. *(B)* Representative fluorescent confocal images of the EBV- Burkitt's lymphoma line BL41 and the EBV+, PTLD-derived B cell lines AB5, JB7, and JC62. *(C)* Representative fluorescent confocal images of the stably transfected BL41.NGFR-LMP1 line, with NGFR-LMP1 crosslinked as described for the indicated amounts of time. For both *(B,C)*, LMP1 stain is shown in red, dAPI stain is shown in blue.

To examine LMP1 signaling we utilized the EBV-negative Burkitt's lymphoma line BL41 and its EBV-infected counterpart BL41.B95, as well as three EBV+ PTLD-derived B cell lines (AB5, JB7, and JC62). All three of the EBV+ PTLD-derived B cell lines express both EBV signaling proteins LMP1 and LMP2a, albeit to different degrees (Figure 1A). BL41.B95, however, expresses LMP1 but minimal, if any, LMP2a protein (Figure 1A). Immunofluorescent staining for LMP1 revealed that native LMP1 is found in aggregates within the membrane in the EBV+, PTLD-derived B cell lines AB5, JB7, and JC62 (Figure 1B), consistent with previous observations in latently-infected B cells [42], [43]. The EBV-negative BL41 cell line served as a negative control for LMP1 expression (Figure 1B).

To study the downstream signaling of LMP1, we utilized the EBV-negative BL41 cell line stably transfected with an NGFR-LMP1 chimeric construct [20], (Figure 1C, and Figure S1A). Chimeric NGFR-LMP1 molecules permit controlled, inducible LMP1 signaling when NGFR is crosslinked as opposed to the constitutive LMP1 signaling characteristic of the native molecule. Expression of native LMP1 increases ICAM (CD54) expression in B cells, T cells, and epithelial cells [17]. Therefore, functionality of crosslinked NGFR-LMP1 was confirmed by the upregulation of ICAM in the stably transfected, but not the parental, BL41 line (Figure S1B). Immunofluorescent staining revealed NGFR-LMP1 is expressed throughout the membrane in stably transfected cells (Figure 1C). Addition of anti-NGFR and crosslinking antibodies resulted in the aggregation of NGFR-LMP1 in the membrane of stably transfected cells (Figure 1C) in a pattern that resembled the expression of native LMP1 molecules (Figure 1B), although we also observe some NGFR-LMP1 expression outside glycosphingolipid (GSL) domains (data not shown).

### NGFR-LMP1 Activates, but Does Not Physically Interact with, Syk

**Figure 2:**
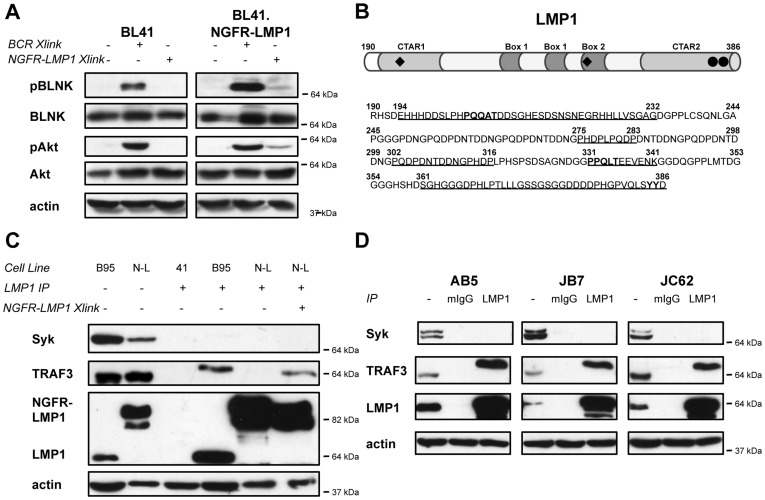
NGFR-LMP1 Activates, but Does Not Physically Interact with, Syk. *(A)* NGFR-LMP1 crosslinking or BCR crosslinking was triggered as described for 5 minutes. Lysates were separated on SDS-PAGE gels, transferred to nitrocellulose, and probed for the indicated phospho-proteins by western blotting. Blots were then stripped and reprobed for total protein levels. *(B)* Schematic and amino acid sequence of the C-terminal signaling tail of LMP1. TRAF binding motifs (PXQXT; black diamonds) and TRADD-interacting tyrosines (Y385, Y385; black circles) are noted in bold. C-terminal activating regions (CTAR), CTAR1 (E194 to G232) and CTAR2 (S361 to D386), are noted in underlined sections and in light grey shading. Potential JAK3 interaction sites, Box1 (P275 to P283, P302 to L316) and Box2 (P331 to K341), are noted in underlined sections and in dark grey shading. *(C)* Cells were treated with anti-NGFR and goat anti-mouse Ig to induce NGFR-LMP1 signaling for 5 minutes as indicated. Lysates from 10×10^6^ cells were immunoprecipitated with anti-LMP1. Washed immunoprecipitates and flow through were separated by SDS-PAGE, transferred to nitrocellulose, and blotted for Syk, TRAF3, LMP1, and actin as a loading control (41 = BL41, B95 = BL41.B95, N-L = BL41.NGFR-LMP1). *(D)* Immunoprecipitation with either mIgG isotype control or anti-LMP1 Abs and western blotting of lysates was performed as described in *(C)*. For all western blots, the closest molecular weight marker (e.g. 64 kDa) to the protein of interest is indicated to the right of each blot.

To assess whether LMP1 signaling activates Syk, we crosslinked NGFR-LMP1 and assayed for phosphorylation of the Syk substrate BLNK. BLNK both directly binds and is phosphorylated by Syk [44], [45]. Addition of anti-NGFR and crosslinking antibodies resulted in phosphorylation of the Syk substrate BLNK in the stably transfected lines, but not the parental BL41 line (Figure 2A, right lanes). Phosphorylation of Akt was used to confirm NGFR-LMP1 crosslinking (Figure 2A, right panel, right lane). Comparatively, NGFR-LMP1 crosslinking elicits less phosphorylation of both BLNK and Akt than BCR crosslinking, however it is able to activate each of these pathways.

Classical Syk activation requires direct binding of the tandem SH2 domains of Syk to either an ITAM in a receptor tail or two hemITAMs on two separate receptor peptide chains. While the C-terminal, cytoplasmic tail of LMP1 contains motifs that bind adaptor molecules like the TRAFs (Figure 2B), it does not contain the canonical tandem Y*XX*L ITAM motif. Given the absence of the canonical ITAM motifs, we asked if LMP1 physically interacts with Syk. Immunoprecipitation of native LMP1 from BL41.B95 did not co-precipitate Syk, but did co-precipitate the adaptor molecule TRAF3 (Figure 2C, B95, lane 4), known to associate with native LMP1. Similarly, immunoprecipitation of NGFR-LMP1 from BL41.NGFR-LMP1 did not co-precipitate Syk, but did co-precipitate the adaptor molecule TRAF3 after NGFR-LMP1 crosslinking (Figure 2C, N-L, lanes 5 and 6). Nevertheless, Syk was present in total cell lysates from BL41.B95 and BL41.NGFR-LMP1 (Figure 2C, lanes 1 and 2). Finally, native LMP1 did not co-precipitate Syk, but did co-precipitate TRAF3 in all three EBV+ PTLD-derived B cell lines (Figure 2D, lane 3). Taken together, these data suggest that while NGFR-LMP1 induces Syk activity, it does so without direct physical interaction with Syk.

### NGFR-LMP1 Activates Syk Upstream of PI3K and IL-10

**Figure 3:**
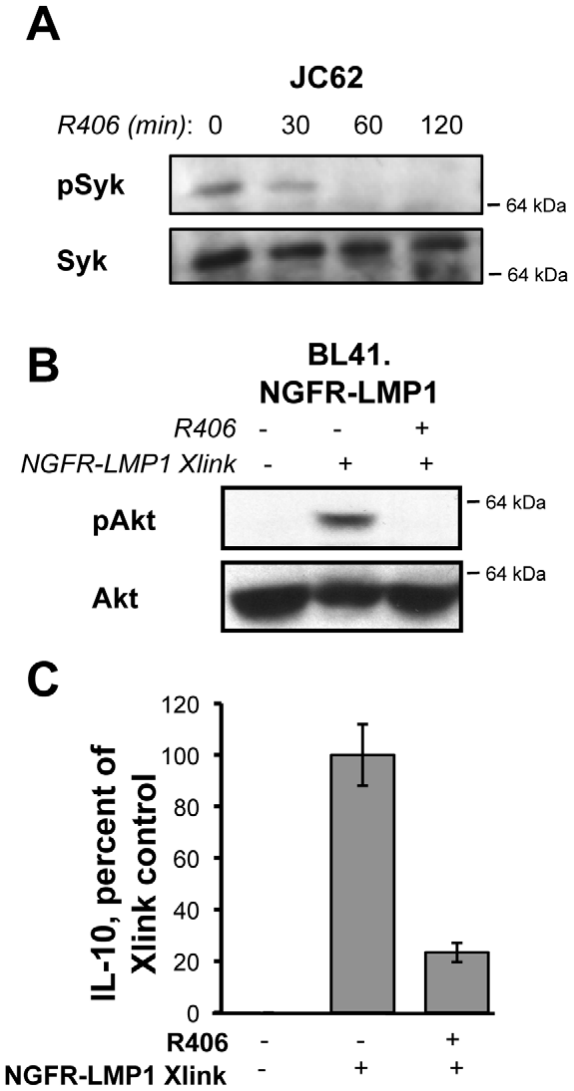
NGFR-LMP1 Activates Syk Upstream of PI3K and IL-10. *(A)* Lysates from R406-treated cells (as indicated) were immunoprecipitated with anti-Syk antibodies. Washed immunoprecipitates were separated by SDS-PAGE, transferred to nitrocellulose, and blotted for pSyk and Syk. JC62 is shown here as a representative cell line. *(B)* NGFR-LMP1 crosslinking was triggered for 5 minutes as indicated, in the presence of DMSO control (−) or the Syk inhibitor R406 (+). Lysates were separated on SDS-PAGE gels, transferred to nitrocellulose, and probed for the indicated proteins by western blotting. The closest molecular weight marker (e.g. 64 kDa) to the protein of interest is indicated to the right of each blot. *(C)* Cells were pretreated for 1 hour with DMSO control (−) or the Syk inhibitor R406 (+) before induction of NGFR-LMP1 signaling. Cells were then plated at 0.5×10^6^/ml in the presence of inhibitor. At 24 hours, supernatants were harvested and assayed for IL-10 by ELISA. IL-10 production was normalized to the number of viable cells at the time of supernatant harvest. Data is expressed as the mean percent of IL-10 produced for each group (compared to control) with standard deviations and is representative of 3–4 replicate experiments.

We next asked if Syk is required for the activation of PI3K by NGFR-LMP1 by utilizing R406, an ATP-competitive inhibitor of Syk. Basal phosphorylation of Syk was detected by immunoprecipitation and western blot of Syk from lysates of EBV+ PTLD-derived B cell lines; a representative blot from the JC62 cell line is shown in Figure 3A. This basal phosphorylation of Syk (Figure 3A, left lane) was reduced when EBV+ PTLD-derived B cell lines were treated with R406 (Figure 3A, right lanes).

Phosphorylation of Akt after NGFR-LMP1 crosslinking (Figure 3B, lanes 1,2) in the stably transfected line is reduced in the presence of the Syk inhibitor R406 (Figure 3B, lane 3). IL-10 is a critical autocrine growth factor for EBV-infected cells, and is required for the autonomous proliferation of EBV+, PTLD derived B cell lines [39]. We have previously shown that IL-10 production by LMP1 is PI3K-dependent [20]. To further establish the role for Syk in PI3K/Akt activation by NGFR-LMP1, we asked if Syk inhibition affects IL-10 production by NGFR-LMP1. Indeed, Syk inhibition reduced NGFR-LMP1-dependent IL-10 production by approximately 80% (Figure 3C). These results suggest that Syk is activated by NGFR-LMP1 upstream of PI3K.

### NGFR-LMP1 Activates Src Family Kinases

**Figure 4:**
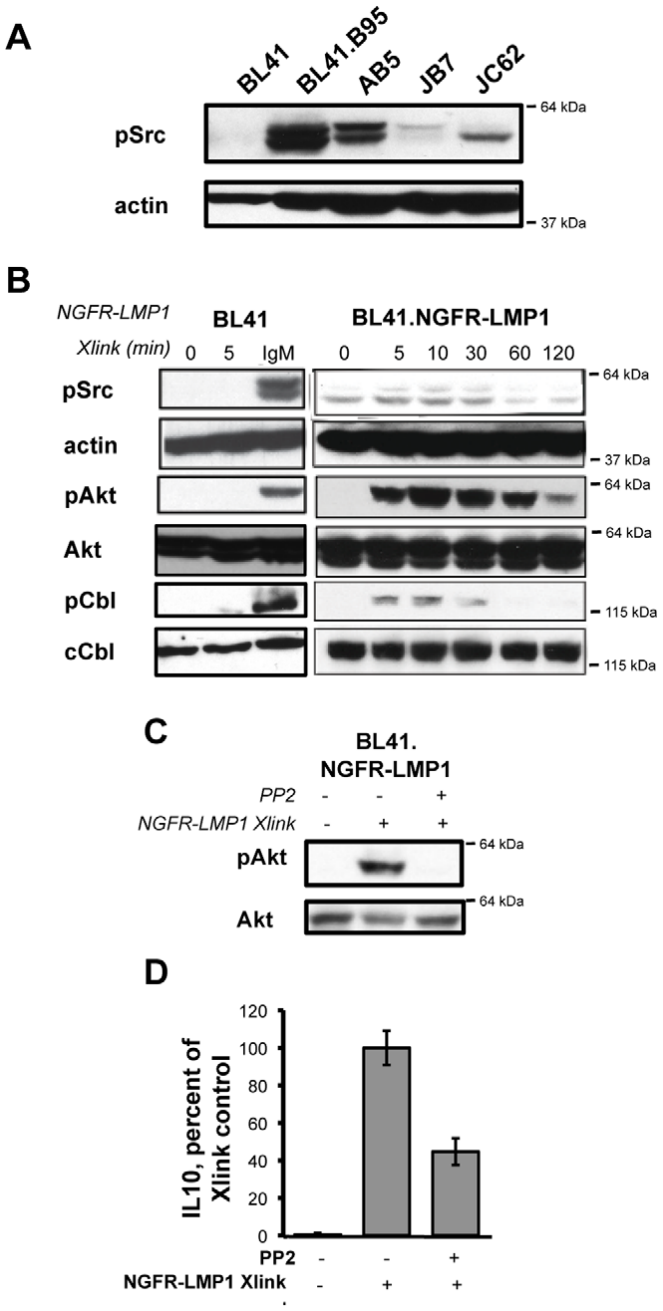
NGFR-LMP1 Activates Src Family Kinases. *(A)* Cell lysates were separated by SDS-PAGE, transferred to nitrocellulose, and probed with indicated antibodies. *(B)* NGFR-LMP1 signaling or BCR signaling (IgM) was triggered for the indicated amount of time. Lysates were separated on SDS-PAGE gels, transferred to nitrocellulose, and probed for the indicated proteins by western blotting. *(C)* Cells were pretreated for 1 hour with DMSO control (−) or the Src inhibitor PP2 (+) before triggering NGFR-LMP1 signaling or BCR signaling for 5 minutes in BL41.NGFR-LMP1 cells as described. Lysates were separated on SDS-PAGE gels, transferred to nitrocellulose, and probed for the indicated proteins by western blotting. For all western blots, the closest molecular weight marker (e.g. 64 kDa) to the protein of interest is indicated to the right of each blot. *(D)* Cells were pretreated for 1 hour with DMSO control (−) or the Src inhibitor PP2 (+) before induction of NGFR-LMP1 signaling. Cells were then plated at 0.5×10^6^/ml in the presence of inhibitor. At 24 hours, supernatants were harvested and assayed for IL-10 by ELISA. IL-10 production was normalized to the number of viable cells at the time of supernatant harvest. Data is expressed as the mean percent of IL-10 produced for each group (compared to control) with standard deviations and is representative of 3–4 replicate experiments.

Classical Syk activation requires the action of Src tyrosine kinases. To determine if Src family kinases also participate in PI3K/Akt activation following NGFR-LMP1 signaling, we first investigated whether Src family kinases are activated in latently infected B cells. Western blot analysis with a pan-specific antibody that recognizes phosphorylated Src family members Src, Lyn, Fyn, Lck, Yes, and Hck, revealed constitutive Src family kinase activation in latently infected cells (Figure 4A). In a time course analysis following NGFR-LMP1 crosslinking, we observed rapid phosphorylation of Src family kinases that coincided with Akt phosphorylation (Figure 4B, right panels). C-Cbl, an E3 ubiquitin ligase often concurrently activated with Src family kinases [46], contains multiple phosphorylation sites required for association with the p85α subunit of PI3K [47]. We observed a rapid, time-dependent phosphorylation of c-Cbl at Tyr700 that peaked 5–10 minutes after LMP1 signaling was initiated (Figure 4B). Inhibition of Src family kinases by the small molecule inhibitor PP2 ablated phosphorylation of Akt by NGFR-LMP1 crosslinking (Figure 4C) and diminished the induction of IL-10 by NGFR-LMP1 crosslinking (Figure 4D). Taken together, these data indicate that NGFR-LMP1 activates Src family kinases upstream of PI3K activation.

### NGFR-LMP1 Activates the Src Kinase Fyn

**Figure 5:**
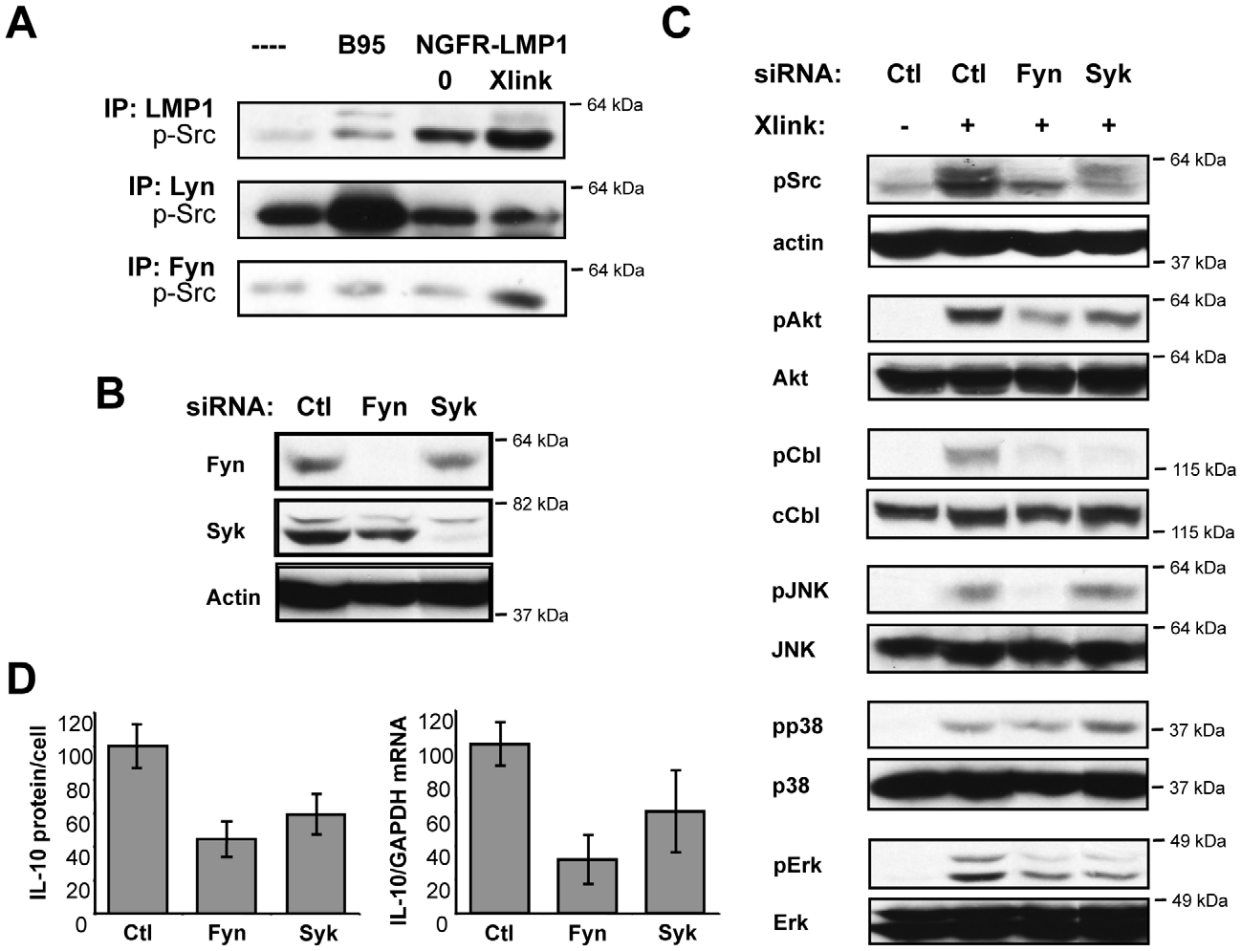
NGFR-LMP1 Activates the Src Kinase Fyn. *(A)* Cell lysates were prepared 15 minutes after the induction of NGFR-LMP1 signaling. Lysates were immunoprecipitated overnight as indicated. Washed immunoprecipitates were separated on SDS-PAGE gels, transferred to nitrocellulose, and probed for phospho-Src by western blotting. *(B–D)* Three million cells were transfected with 200 nM RNAi specific for either Fyn or Syk 96 hours prior to analysis. *(B, C)* Knockdown confirmation and downstream signaling analysis. NGFR-LMP1 signaling was triggered in RNAi transfected cells for 30 minutes. Lysates were separated on SDS-PAGE gels, transferred to nitrocellulose, and probed for the indicated proteins by western blotting. For all western blots, the closest molecular weight marker (e.g. 64 kDa) to the protein of interest is indicated to the right of each blot. *(D)* IL-10 production. RNAi transfected cells were treated with anti-NGFR and goat anti-mouse Ig. At 24 hours, supernatants were harvested and assayed for IL-10 by ELISA. IL-10 cytokine production was normalized as previously described, with data expressed as the mean percent of IL-10 produced for each group (compared to Xlink control group) with standard deviations and represents the combined results of 3–4 replicate experiments. IL-10 mRNA expression was evaluated in parallel cultures by quantitative RT-PCR following 18 hours of NGFR-LMP1 signaling. Data is expressed as the mean percent of IL-10/GAPDH for each group (compared to the control group) with standard deviations and represents the combined results of 2 experiments.

The Src family tyrosine kinase inhibitor PP2 can inhibit all of the Src family kinases - Src (p60), Fgr (p58), Fyn (p59), Hck (p59/p61), Lck (p56), Lyn (p53/p56), Yes (p62), and Yrk (p60). Additionally, the phosphorylated forms of Src, Fyn, Hck, Lyn, and Yes are each recognized by the anti-phospho-Src family antibody. Thus, it was important to determine the specific Src family kinase phosphorylated by NGFR-LMP1 crosslinking. We first determined whether any phosphorylated Src family kinase member could directly associate with native LMP1 or chimeric NGFR-LMP1. We found that a phosphorylated Src family kinase of approximately 55–60 kDa co-precipitated with LMP1 (Figure 5A, top panel). This Src family kinase co-precipitated with native LMP1 in EBV-infected BL41.B95 cells (Figure 5A top panel, lane 2) and its association with NGFR-LMP1 was increased upon NGFR-LMP1 crosslinking (Figure 5A, top panel, lanes 3 and 4).

We then analyzed the activation of specific Src family kinases by immunoprecipitation and western blot. Based on the molecular weight of the LMP1-associated protein and their known expression in B cells, Lyn and Fyn were the mostly likely Src family members. Immunoprecipitated Lyn was hyper-phosphorylated in EBV-infected BL41.B95 cells (Figure 5A, middle panel, lane 2), but NGFR-LMP1 crosslinking did not increase Lyn phosphorylation in BL41.NGFR-LMP1 cells (Figure 5A, middle panel, lanes 3 and 4). These data indicate that Lyn is likely not the Src family kinase involved in LMP1 signaling. In contrast, immunoprecipitated Fyn kinase was found to be hyper-phosphorylated in BL41.NGFR-LMP1 cells only after induction of NGFR-LMP1 signaling (Figure 5A, bottom panel, lanes 3 and 4). Taken together, these results strongly suggest that Fyn is the Src family kinase involved in activation of PI3K by NGFR-LMP1.

### NGFR-LMP1 Activates Fyn Upstream of PI3K and IL-10

To confirm the role of Syk and Fyn in NGFR-LMP1 activation of PI3K, we utilized siRNA to knockdown Syk and Fyn in NGFR-LMP1 expressing cells. We routinely achieved knockdown of ∼90% for Fyn and ∼80% for Syk compared to the siRNA control in BL41.NGFR-LMP1 cells (Figure 5B). Fyn knockdown also decreased the levels of phospho-Src in NGFR-LMP1-crosslinked cells (Figure 5C), consistent with the interpretation that Fyn is the upstream Src kinase involved in LMP1 signaling. Syk knockdown did not affect the levels of phospho-Src in NGFR-LMP1-crosslinked cells (Figure 5C), suggesting that Syk is downstream of the Src family kinases.

In concordance with our inhibitor data, at least a 50% decrease in NGFR-LMP1-dependent Akt phosphorylation was observed in cells in which either Syk or Fyn was knocked down (Figure 5C). As the downstream molecules of interest (Akt, c-Cbl, Jnk, p38, and Erk) are not activated without NGFR-LMP1 crosslinking (Figure 5C, left lanes), we used crosslinked cells treated with control siRNA as our negative control for these experiments. Knockdown of either Syk or Fyn decreased NGFR-LMP1-dependent IL-10 cytokine secretion by ∼40% and ∼60%, respectively (Figure 5D, left panel). Fyn or Syk knockdown also inhibited NGFR-LMP1-dependent IL-10 mRNA expression (Figure 5D, right panel) in agreement with our earlier published results that LMP1 regulates IL-10 transcription [20]. Taken together with the previous data, these findings demonstrate that both Fyn and Syk play essential roles in NGFR-LMP1 signaling upstream of PI3K.

As discussed previously, we observed rapid c-Cbl phosphorylation following NGFR-LMP1 crosslinking (Figure 4B). Since both Fyn and Syk are known to be c-Cbl kinases, and since phosphorylated c-Cbl can interact with the p85α subunit of PI3K [48], we asked whether NGFR-LMP1-dependent c-Cbl phosphorylation levels correlated with NGFR-LMP1-dependent Akt phosphorylation levels. Indeed, in both the Fyn knockdown and the Syk knockdown cells, decreased Akt phosphorylation was accompanied by significantly decreased c-Cbl phosphorylation (down 70–80%) (Figure 5C). These data indicate that both Fyn and Syk activation are required for maximal phosphorylation of c-Cbl following NGFR-LMP1 signaling.

LMP1 is also a potent inducer of the MAPK pathways in EBV-infected B cells but the specific signaling intermediates that participate in MAPK activation have not been determined. Interestingly, our results reveal a difference in the requirements for Fyn and Syk in the NGFR-LMP1-dependent activation of MAPK. Whereas maximal JNK activation required Fyn but not Syk (Figure 5C), p38 activation by NGFR-LMP1crosslinking was not dependent on either Fyn or Syk (Figure 5C). In contrast, Fyn and Syk were each required for optimal Erk activation following NGFR-LMP1 crosslinking (Figure 5C). It has been previously reported that, during TCR signaling, Fyn activates the Erk MAPK pathway [49], but to our knowledge this is the first indication of Src-dependent induction of Erk MAPK by LMP1 in any cell type.

## Discussion

EBV is associated with several lymphoid malignancies including Hodgkin's disease, Burkitt's lymphoma, and PTLD. Whereas the EBV protein LMP1 activates the oncogenic PI3K/Akt signaling pathway, the mechanism of PI3K/Akt activation is of interest for unveiling virally-controlled therapeutic targets for the treatment of EBV-associated malignancies. Using the inducible NGFR-LMP1 protein as a model system to study LMP1 signaling, we describe here the first evidence for non-canonical Syk activation in B cells by the EBV oncogene LMP1. Additionally, we provide the first evidence for activation of Src family kinases, in particular Fyn, as well as activation of c-Cbl, in B cells by LMP1. By demonstrating a requirement for Syk and Fyn in LMP1-induced PI3K/Akt, c-Cbl, and IL-10 induction, we also gained insight into the signaling mechanism by which LMP1 induces PI3K-dependent Akt phosphorylation in B cells.

Several lines of evidence indicate that the NGFR-LMP1 inducible chimeric protein is a useful model system to study LMP1 signaling. In particular, NGFR-LMP1 has replicated each signaling event and functional outcome associated with native LMP1 that we have tested. First, native LMP1 associates with TRAF1 and TRAF3 (Figure 2D and [7]–[9]). Similarly, TRAF1 and TRAF3 associate with NGFR-LMP1 after crosslinking (Figure 2C and [20]). Second, native LMP1 activates the following signaling pathways: PI3K/Akt [21], Erk [12], [13], p38 [10], [11], JNK [14], [15], and NF-kB [16], [17]. Similarly, each of these pathways is activated after NGFR-LMP1 crosslinking (Figures 2A, 3B, 4B, 4C, 5C and [20], [50]). Thirdly, native LMP1 induces the upregulation of ICAM (CD54) [17] and induces the production of IL-10 [11], a growth factor required for the autonomous proliferation of EBV+ PTLD-derived B cell lines [39]. Similarly, NGFR-LMP1 crosslinking induces the upregulation of ICAM (Figure S1B and [50]) and the production of IL-10 (Figures 3C, 4D, 5D and [20]). Finally, upon crosslinking, NGFR-LMP1 aggregates within the membrane in a manner similar to native LMP1 (Figures 1B, 1C). Together, these findings strongly support that the results obtained using the NGFR-LMP1 fusion protein are pertinent to native LMP1.

Our finding that LMP1 activates Syk sheds light on the mechanism of constitutive Syk activation observed in EBV-infected B cells, which had previously been attributed exclusively to LMP2a. Since LMP1 and LMP2a co-localize in the plasma membrane in latently-infected cells [43], they are spatially arranged to cooperate with each other. Indeed, there is precedence for cross-talk between these two proteins because LMP2a enhances LMP1-driven NF-κB and AP-1 activation [51]. Similarly, Syk activation in EBV-infected B cells may be a cooperative effort between LMP1 and LMP2a. Given that LMP1 and LMP2a are the viral homologs of CD40 and the BCR, respectively, our data suggests a more generalized function for non-canonical activation of Syk in B cells. Indeed, recent evidence suggests that CD40 can activate members of the BCR signaling complex, like BLNK, through Syk activation, resulting in enhanced BCR signaling [52]. Understanding the mechanism of Syk activation by LMP1 may yield more general insights into CD40 function and its modulation of BCR signaling.

We observed activation of Syk by LMP1 in the absence of physical interaction. Studies of Syk activation by integrins and P-selectin glycoprotein ligand 1 (PSGL1) have generated four potential models for non-canonical Syk activation [25]. The first, an ITAM-independent model, involves the interaction of Syk with the integrin β-chain, and the subsequent activation of Syk by Src family kinases. In the ITAM-mediated model, integrin ligation triggers the phosphorylation of ITAM-bearing signaling adaptors DAP12 or FcRγ by Src family kinases, resulting in the recruitment and activation of Syk. The third model involves a combination of the first two models, with Syk binding both integrins and the Src family-phosphorylated ITAMs of a signaling adaptor. In the fourth model, PSGL1 activation results in phosphorylation of the ITAMs exrin, radixin, and moesin (ERM) family proteins, which recruit and activate Syk [53]. Given that we observed no direct physical interaction between LMP1 and Syk, it is likely that the ITAM-independent model is not involved. Closer examination into the role of ITAM-containing signaling proteins, like ERM family proteins, and adaptor proteins, like DAP12 and FcRγ, is warranted to better understand the mechanism of Syk activation by LMP1.

Common among the potential mechanisms of non-canonical Syk activation is the involvement of Src family kinases. Indeed, our data shows activation of Src family kinases, specifically Fyn, by LMP1. Fyn also phosphorylates another viral protein reminiscent of LMP1 – K15 of Kaposi's sarcoma herpes virus [54]. Fyn is only one of multiple Src family kinases expressed in B lymphocytes. Depending on the receptor and signaling adaptor involved, different Src family kinases are activated. For example, while our data indicates LMP1 activates Fyn, the BCR and LMP2a primarily utilize Lyn, and DAP12/FcRγ-mediated PSGL1 signaling involves Fgr activation [55]. The unique contribution of different Src family kinases to Syk activation and the subsequent cellular response is of particular interest for further investigation. Fyn is more typically associated with function in T lymphocytes, where Fyn activation by the TCR results in recruitment and activation of Syk [56], [57]. Given the role of Fyn in Syk activation by the TCR, it is likely that Fyn activation by LMP1 results in the subsequent activation of Syk. However, further examination of the involvement of Fyn in Syk activation by LMP1 is warranted.

We show that LMP1 signals through PI3K in a manner at least partially dependent on Fyn and Syk. Both Syk and Fyn are reported c-Cbl kinases. Syk can phosphorylate c-Cbl at tyrosines 700, 731, and 774 with equal affinity [47] while Fyn phosphorylates c-Cbl preferentially at tyrosine 731 [58]. Our observation that LMP1 activation results in c-Cbl phosphorylation provides insight into the mechanism by which LMP1 induces PI3K-dependent Akt phosphorylation in B cells. Both Fyn- and Syk- phosphorylated c-Cbl strongly associate with the p85α subunit of PI3K to the same degree *in vitro* [47]. Fyn and Syk act together in T cells to phosphorylate c-Cbl and activate PI3K [56], [57]. In B cells, CD40, the cellular homolog of LMP1, is reported to activate PI3K through involvement of the Src family kinase Lyn, c-Cbl, and p85α [59]. More recent reports on c-Cbl suggest that it may also act as a regulator of PI3K activation through targeted ubiquitination and degradation of associated molecules. For instance, Syk has been reported as a target of c-Cbl mediated ubiquitination following BCR signaling [60], as well as following FcεRI signaling [61]. Whether the positive or the negative regulatory aspects of c-Cbl phosphorylation are necessary for LMP1 activation of the PI3K pathway is one that should be explored in future studies.

We also observed that Fyn and Syk activation by LMP1 had effects on MAPK pathways. Fyn kinases have been reported to stimulate the Erk pathway in TCR signaling [49]. In LMP1 signaling, we observed that both Fyn and Syk are involved in activating the Erk pathway, providing the first evidence for involvement of Src-family kinases in the activation of Erk by LMP1. Additionally, Fyn appears to be involved in activation of the JNK pathway. However, neither Syk nor Fyn plays any role in p38 activation. Such distinct activation requirements upstream of similar MAPK pathways may contribute to the bifurcation of signaling outcomes.

In summary, our data demonstrate the requirement for activation of Syk and Fyn by LMP1 upstream of PI3K. Understanding how an oncoprotein such as LMP1 activates Src family kinases, Syk and PI3K has implications for treatment of EBV+ B cell lymphomas.

## Supporting Information

Figure S1 **Inducible LMP1 Signaling.** *(A)* Stable NGFR-LMP1 expressing lines and the parental BL41 line were stained with biotinylated anti-NGFR followed by streptavadin-PE to assess surface expression levels of the NGFR-LMP1 construct. *(B)* Cells were treated with anti-NGFR and goat-anti mouse Ig to induce NGFR-LMP1 signaling for 18 hours. After excess crosslinking antibody was neutralized by the addition of mouse IgG isotype control antibodies, cells were stained with ICAM-PE to assay for NGFR-LMP1 functionality.
(TIF)
