# Supplementary figures and images for: Src Kinase and Syk Activation Initiate PI3K Signaling by a Chimeric Latent Membrane Protein 1 in Epstein-Barr Virus (EBV)+ B Cell Lymphomas
Source: PLoS One. 2012 Aug 3;7(8):e42610. doi: 10.1371/journal.pone.0042610 (PMC3411813; doi:10.1371/journal.pone.0042610)

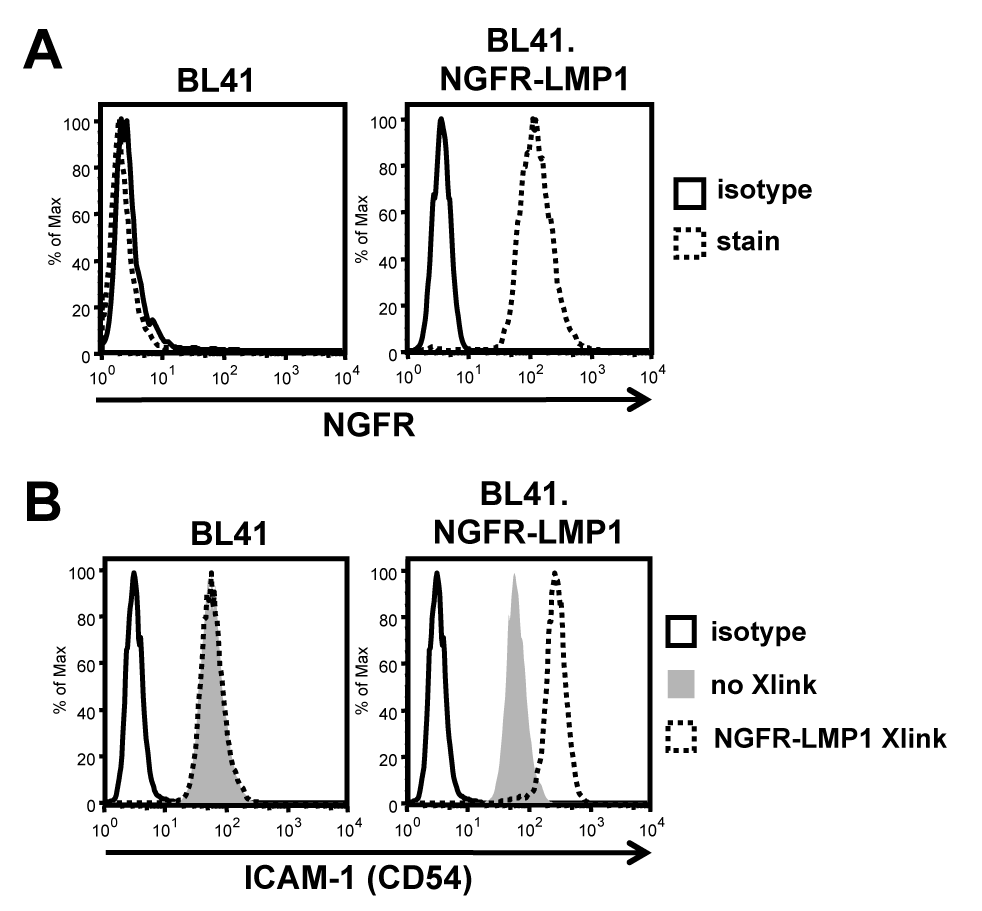

Supplement: Figure S1 — Inducible LMP1 Signaling. (A) Stable NGFR-LMP1 expressing lines and the parental BL41 line were stained with biotinylated anti-NGFR followed by streptavadin-PE to assess surface expression levels of the NGFR-LMP1 construct. (B) Cells were treated with anti-NGFR and goat-anti mouse Ig to induce NGFR-LMP1 signaling for 18 hours. After excess crosslinking antibody was neutralized by the addition of mouse IgG isotype control antibodies, cells were stained with ICAM-PE to assay for NGFR-LMP1 functionality. (TIF) [file pone.0042610.s001.tif]
